# Recent Synthetic Biology Approaches for Temperature- and Light-Controlled Gene Expression in Bacterial Hosts

**DOI:** 10.3390/molecules27206798

**Published:** 2022-10-11

**Authors:** Jongdoo Choi, Jiyeun Ahn, Jieun Bae, Minseob Koh

**Affiliations:** Department of Chemistry, Pusan National University, Busan 46241, Korea

**Keywords:** thermosensor, photocaged inducer, photoswitch, genetic code expansion, optogenetics

## Abstract

The expression of genes of interest (GOI) can be initiated by providing external stimuli such as temperature shifts and light irradiation. The application of thermal or light stimuli triggers structural changes in stimuli-sensitive biomolecules within the cell, thereby inducing or repressing gene expression. Over the past two decades, several groups have reported genetic circuits that use natural or engineered stimuli-sensitive modules to manipulate gene expression. Here, we summarize versatile strategies of thermosensors and light-driven systems for the conditional expression of GOI in bacterial hosts.

## 1. Introduction

Organisms have evolved the ability to sense external stimuli, including changes in temperature and light irradiation. Temperature affects the growth rate and pathogenesis of certain microbes [1]. External temperature changes may trigger cellular mechanisms involving homeostasis, such as the chaperone system, which is responsible for protein folding [2], and virulence genes of pathogenic bacteria, which promote survival mechanisms when they are in the host [3]. Light sources, however, can be directly utilized as energy sources in the photosynthetic processes of plants, algae, and cyanobacteria [4]. In addition to this, organisms have evolved to sense light, as exemplified by vision [5].

**Figure 1:**
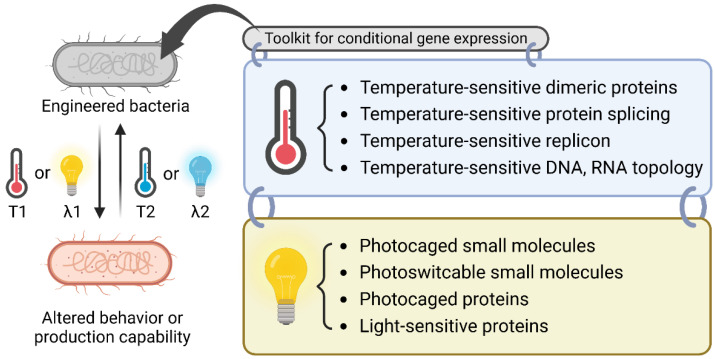
Overview of the molecular biology toolkit for conditional gene expression. A bacterium can be engineered to be sensitive to a temperature shift or specific light irradiation for the dynamic regulation of bacterial behavior or the production of chemicals or proteins. The toolkit for thermoregulation includes temperature-sensitive dimeric proteins, protein splicing, replicons, and oligonucleotide topology. For light-sensitive regulation, one can choose from the toolkit of photocaged small molecules, photoswitchable small molecules, photocaged proteins, and light-sensitive proteins. T1, T2: different temperatures; λ1, λ2: different wavelengths.

From a synthetic biology perspective, nature’s mechanisms for sensing temperature and light have provided powerful insights for designing tools to achieve conditional gene expression. At the molecular level, researchers have utilized the heat shock response mechanism to construct temperature-sensitive gene expression systems such as the temperature-sensitive dimeric repressor [6,7,8,9,10,11,12,13], RNA polymerase (RNAP) [14,15,16,17], replication machinery [18,19,20,21,22], and oligonucleotide topology [23,24,25,26,27,28,29,30,31,32,33]. Photocaged [34,35,36,37,38,39,40,41,42,43] or photoswitchable [44,45] small-molecule inducers and photocaged transcription factors [46] have been used to develop light-driven gene expression systems. Additionally, light-sensitive proteins have been widely used as essential modules to control diverse cellular functions in optogenetics [5] (Figure 1).

In this review, we discuss recent advances in synthetic biology that utilize temperature- and light-sensitive modules for conditional gene expression in bacterial hosts.

## 2. Temperature-Controlled Gene Expression

The induction of gene expression by temperature has many advantages, including being low-cost, requiring simple instrumentation, being non-invasive, having minimal contamination risk, and being reversible. Diverse regulatory systems in cells that utilize proteins, RNA, and DNA are responsible for sensing temperature inputs [1,47,48]. In this section, we provide the temperature-sensing modules found in nature and discuss how they can be utilized in genetic circuit design.

### 2.1. Engineering of Dimeric Repressor

**Figure 2:**
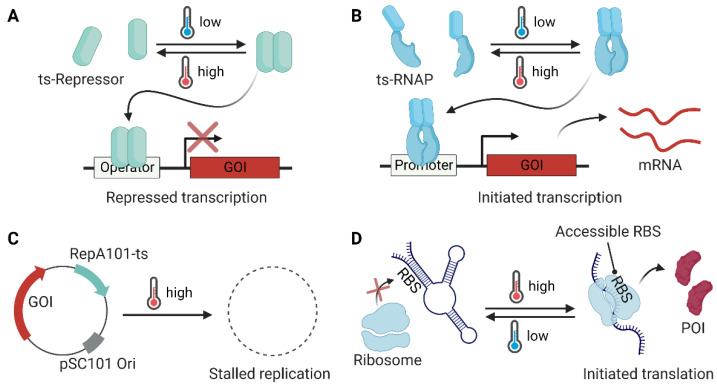
Temperature-sensitive modules utilized in bacterial engineering. (**A**) A temperature-sensitive (ts) dimeric repressor (e.g., cI857 and TlpA) forms a dimer and binds to the operator, thereby inhibiting gene expression at low temperatures. (**B**) A temperature-sensitive dimer module (e.g., the coiled-coil domain of TlpA) can be fused to split RNA polymerase (RNAP) to generate a temperature-sensitive transcription system. (**C**) A temperature-sensitive replication system restricts the replication of a plasmid that contains the genes of interest (GOI) at the defined temperature. (**D**) An RNA thermometer can be designed to have a buried ribosome binding site (RBS) by forming a stable hairpin structure at low temperatures (<30 °C) to inhibit translation, whereas the RBS is exposed at high temperatures (>37 °C) for ribosome entry and the translation of the GOI to generate the protein of interest (POI).

The bacteriophage lambda cI homodimer binds to its cognate operator sites (oR1, oR2, and oR3) in the pR/pRM promoter [49]. In the bacteriophage, the wild-type cI is temperature-insensitive, and the induction of downstream gene expression is enabled by the RecA-mediated autocatalytic cleavage of cI in the lytic growth stage [50]. The mutant protein cI857 gains temperature sensitivity due to unstable dimer formation at higher temperatures (>37 °C), caused by the Ala66Thr mutation at the N-terminus [51] (Figure 2A). The pL/pR-cI857 thermoinduced expression system is useful for the expression of heterologous proteins [52] in phages, viruses, and humans as well as high-value metabolites [10] in *Escherichia coli*. In addition, the cI857 repression system is useful in constructing genetic circuits for bifunctional dynamic control [7] and kill switches [13]. cI857 variants can also be used for temperature-sensitive transcriptional activation [6]. Recently, a genetic circuit for a cold-inducible switch was developed [11]. A cold-inactivated variant of the bacteriophage 434 cI repressor containing a TEV recognition site was coupled with a heat-inactivated TEV protease and a *Mycoplasma florum* Lon protease for tight control.

Another useful dimeric repressor is TlpA from Salmonella [53], which possesses an intrinsic temperature sensitivity in the formation of its coiled-coil structure. TlpA binds to its cognate promoter and represses downstream gene expression at temperatures below 37 °C, whereas its structural instability at higher temperatures (>42 °C) confers a loss of repression [47] (Figure 2A). Recently, the Shapiro group reported engineered TlpA variants that shifted working temperatures relative to the wild-type. They also developed similar versions of the cI857 variants to produce orthogonal thermal bioswitches. It is noteworthy that such a fine-tuned thermal switch can be utilized for the spatiotemporal control of microbial therapeutics using focused ultrasound [12].

### 2.2. Regulation of RNAP

RNAP can be engineered directly to achieve temperature sensitivity. Liang et al. adopted a temperature-sensitive *Saccharomyces cerevisiae* VMA intein (intein^TS^) [17] to engineer temperature-sensitive T7 RNAP for conditional gene expression at 18 °C in *E. coli* [16]. The intein^TS^ fused in the middle of the T7 RNAP (between Ala491 and Cys492) can be cleaved by an autonomous protein splicing process and can generate full-length T7 RNAP only at a low temperature (18 °C). The same idea was utilized in metabolic engineering for lycopene biosynthesis in *E. coli*, which resulted in a 15% improvement in productivity [15]. Recently, the Poh research group reported a reversibly controlled temperature-sensitive gene expression system by the fusion of the C-terminal coiled-coil domain of TlpA (Ala94–Ala371 region, 278 amino acids, TlpA coil) with split-T7 RNAP. To generate the Thermal T7RNAP, N-terminal fragment of T7 RNAP (Met1–Ser564)–TlpA coil and TlpA coil–C-terminal fragment of T7 RNAP (Glu565–Ala883) fusions were used [14] (Figure 2B).

### 2.3. Conditional Replication of Plasmid

Conditional gene expression was achieved using a plasmid containing a temperature-sensitive replicon. The temperature-sensitive plasmid pSC101-ts has been widely used for conditional gene expression at low temperature (30 °C) in *E. coli* [22,54]. The mutation (Ala56Val) in the RepA protein, which is responsible for the replication initiation of pSC101 [55], confers temperature sensitivity to plasmid replication [56] (Figure 2C). The temperature-sensitive replicon of pSC101-ts has been utilized in many recombineering efforts [18,21]. Recently, efficient gene inactivation in *Pseudomonas aeruginosa* was reported using a low-temperature (30 °C) adopted replicon [19], which contains Gly100Cys and Ser204Arg mutations in the replication protein [20].

### 2.4. Regulation of Oligonucleotide Topology

#### 2.4.1. RNA Thermometer (RNAT)

Thermosensing using RNA has advantages over other biomolecular tools for temperature-sensitive gene expression. Plausible reasons may include the quick rearrangement of its secondary and tertiary structures upon temperature change. To regulate the expression of pathogenesis-related genes and heat shock genes, bacteria utilize an RNA-based thermoregulation system [47], such as the ROSE element (repression of heat shock gene expression element) [57]. The Shine–Dalgarno (SD) region of the mRNA is buried at low temperatures and exposed at higher temperatures. At high temperatures, the downstream gene can be expressed (Figure 2D). Synthetic RNATs have been reported, which localize anti-SD sequences upstream of the SD region for heat-induced gene expression [27] or repression [26]. RNATs can also be utilized in the design of temperature-responsive transcription terminators [24] and temperature-sensing protocells [23].

The conditional expression of sigma factor 32 (σ^32^) is also regulated by an RNAT. The mRNA of the σ^32^ coding gene (*rpoH*) has a distinct secondary structure, where the SD sequence and AUG start codon are inaccessible to the 30S ribosome for translation at low temperatures [58]. Elevated temperatures promote the unfolding of such inhibitory structures and facilitate ribosome binding for σ^32^ expression in response to heat stimuli [59]. The three-way junction of the highly structured mRNA of *rpoH* can be targeted by small-molecule stabilizers to disrupt the temperature-sensing mechanism of bacteria [25].

#### 2.4.2. Histone-Like Nucleoid Structuring Protein (H-NS)

H-NS, a chromatin-structuring protein in bacteria, responds to environmental temperature changes. In total, 69% of the temperature-regulated genes of *E. coli* K-12 [32] and 77% of thermoregulated genes of *Salmonella typhimurium* [33] are associated with H-NS. H-NS binds to a specific site on DNA and forms higher-order oligomers, which cause DNA bending and inhibit RNA polymerase access. The oligomerization status of H-NS is temperature-dependent. Gene transcription is re-initiated when bacteria are exposed to a permissive temperature [60]. In *E. coli* K-12, genes related to nutrient acquisition are highly expressed at 37 °C, while genes related to stress response, biofilm formation, and cold shock are highly expressed at 23 °C. This is regulated by H-NS [32]. The molecular mechanism of H-NS de-oligomerization at low temperatures remains largely unknown. However, the regulation of the post-translational modification of H-NS may contribute to gene expression at low temperatures, following a similar mechanism as the xenogeneic silencing of *Shewanella oneidensis* [28]. Despite its versatile use in nature, there have been no successful applications of H-NS in synthetic biology. This is possibly due to the challenges in finding an orthogonal system relative to the other strategies described in this review.

## 3. Light-Driven Regulation of Gene Expression

Light is the perfect choice as a stimulus for inducing gene expression because it is non-invasive, highly controllable, orthogonal, and precise, and can be used to achieve spatiotemporal control [5]. In this section, transcription systems using light-sensitive small molecules or proteins and optogenetic systems using photosensitive proteins, focusing on the flavin-binding light oxygen and voltage (LOV) domains, are discussed.

### 3.1. Photocaged Small-Molecule Inducers

**Figure 3:**
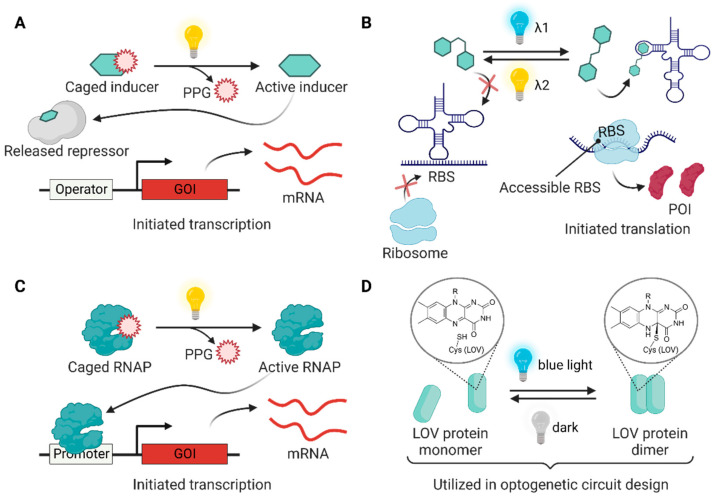
Light-driven regulation modules utilized in bacterial engineering. (**A**) Chemical inducers can be inactivated by installing a photocleavable protecting group (PPG) and activated by irradiating light to unmask the PPG for the induction of gene expression. (**B**) A photoswitchable small molecule (e.g., stiff-stilbene and azobenzene) non-covalently binds to riboswitches, leading to the reversible expression of GOI under the controlled exposure of two different light signals. (**C**) A genetically encoded photocaged non-canonical amino acid (ncAA) allows the construction of photocaged RNAP, whose function can be triggered by light for GOI expression. (**D**) Some LOV domains (e.g., VVD, EL222, and Magnets) dimerize when flavin cofactor forms a covalent bond with the active-site cysteine under blue light irradiation. In the dark, the structures return to their original state through thermal processes. The photocycle of the LOV domains can be utilized in optogenetic circuit design.

To address the low spatiotemporal precision of small-molecule inducers, caging strategies have been used to activate gene expression at specific times and regions [61]. The activity of the caged inducers is initially inhibited and is regained upon light irradiation at the appropriate wavelength (Figure 3A). Small-molecule inducers such as isopropyl *β*-d-1-thiogalactopyranoside (IPTG) [34,35,36,38,39,42,43] and l-arabinose [34,40] are usually targeted for caging. Among the collection of many photocleavable protecting groups (PPGs) [62], limited caging groups including *ortho*-nitrobenzyl (ONB) derivatives and coumarin derivatives have been employed to develop photocaged small-molecule inducers. Caged IPTG [43] was utilized for light-triggered gene expression at the single-cell level [42] for the production of the commodity compound (+)-valencene [39], for a parallelized gene expression setup [38], and for optochemical control of the microbial consortium [36]. In addition to the caged inducer, gene expression can also be triggered by the caged riboswitch systems, such as the caged synthetic riboswitch ligand theophylline [41,63] and caged guanine [37] for photocontrolled gene expression in *E. coli*.

### 3.2. Photosensitive Small-Molecule Switch

One of the limitations of light sensing with photocaged small molecules is their irreversibility. Once the PPG is deprotected, the forward reaction cannot be stopped, and the reverse reaction cannot be induced. Photoswitchable molecules have two isomeric states that can be interconverted through irradiation with different light inputs at different wavelengths [64], which enables reversible genetic control. Although challenging, controlling gene expression with a photoswitchable riboswitch ligand [65,66] offers the opportunity to reversibly regulate artificial genetic circuits [67]. Recently, in vivo demonstrations of stiff-stilbene- [45] and azobenzene-based [44] photoswitchable ligands were reported, showing the regulation of RNA-controlled gene expression in *E. coli* (Figure 3B).

### 3.3. Photocaged Transcription Factor

Advances in genetic code expansion strategies have enabled the incorporation of a non-canonical amino acid (ncAA) to the tailored site of proteins in vivo [68]. A requirement of this strategy is an orthogonal amino acyl tRNA synthetase/tRNA pair that lacks cross-reactivity with the host translational machinery [69]. Among the large libraries of ncAAs that are encoded in the genome, photocaged amino acids and azobenzene-containing amino acids have been reported in many photoresponsive bioapplications [70]. In 2010, the Deiters research group reported photocaged T7 RNAP for light-activated gene expression in *E. coli*. The Tyr639 position of T7 RNAP was mutated to ONBY, the ONB-protected tyrosine, to inactivate T7 RNAP by inhibiting the NTP binding site. The irradiation of 365 nm UV removed the ONB group and restored T7 RNAP function for gene expression [46] (Figure 3C).

### 3.4. Engineering Light-Sensitive Proteins

Recently developed optogenetic approaches commonly utilize natural or engineered photoreceptor-regulated systems because they are highly reversible and genetically encodable without additional synthetic components [5]. The essential chromophores of photoreceptors include tryptophan (in UVR8 [71]), flavins (in LOV domains [72], BLUF domains [73], and cryptochromes [74]), cobalamins (in cobalamin-binding domains [75]), and tetrapyrroles (in phytochromes [76]).

LOV domains have higher versatility in circuit design for bacterial gene expression compared to other photoreceptors due to their small sizes and naturally available flavin chromophores [77]. LOV domains are classified in the PAS (period circadian protein–aryl hydrocarbon receptor nuclear translocator protein–single-minded protein) [78,79] family. When irradiated with blue light, a thioether bond between flavin and a nearby cysteine residue of the PAS core is formed, resulting in structural changes in the LOV domain [72,77]. The light-induced structural changes reverted to the ground state through the thermal relaxation process in the dark [80] (Figure 3D).

In this subsection, we review how LOV domains are utilized in optogenetic circuit designs in bacteria by categorizing them into two-component and one-component systems.

#### 3.4.1. Two-Component Systems

**Figure 4:**
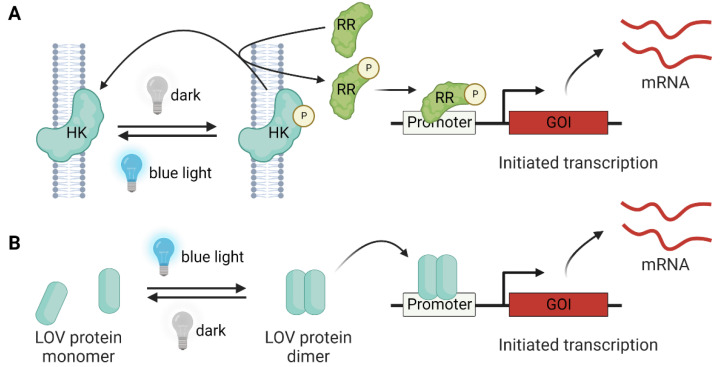
Light-controlled gene expression by LOV proteins. (**A**) The two-component system consists of histidine kinase (HK) and a response regulator (RR) for signal relay. In the absence of light, the HK is phosphorylated, and the phosphate group is transferred to the RR, leading to GOI expression. The blue light induces the dephosphorylation of HK and RR, and the GOI expression is inhibited. (**B**) In the one-component system, the blue-light-driven LOV dimerization (e.g., EL222) can directly trigger GOI expression.

In a two-component system (TCS), for example, the blue-light-regulated LOV-domain-containing histidine kinase (HK) is autophosphorylated and transfers a phosphate group to a cognate response regulator (RR), which in turn triggers the expression of the corresponding gene [81] (Figure 4A). In 2012, Ohlendorf et al. reported a pDusk/pDawn system based on HK YF1 and RR FixJ for light-driven gene expression [82]. In the dark, YF1 [83] phosphorylates FixJ, resulting in gene expression from the FixK2 promoter. The above process is largely hampered by blue light irradiation, and the expression of genes of interest (GOI) is repressed (pDusk). Similarly, GOI expression can be activated by light (pDawn) by inserting the cI repressor and lambda promoter pR into the pDusk system. The pDusk/pDawn system is a widely used LOV-based TCS. For example, pDawn has been used in biofilm lithography with *E. coli* [84], light-induced biofilm inhibition in *P. aeruginosa* [85], the production of drug-releasing living material [86], and the optogenetic control of the *lac* operon (OptoLAC) for chemical and protein production in *E. coli* [87]. Recently, the Avalos research group reported a light-regulated toxin–antitoxin system (OptoTA) using the pDusk/pDawn system for the dynamic regulation of bacterial populations [88].

#### 3.4.2. One-Component Systems

One-component systems (OCSs) are relatively faster than TCSs in reversible signaling because of the more direct and simpler designs of the genetic circuits [81] (Figure 4B). The OCSs utilized in bacteria largely rely on LOV-domain-containing modules, including VVD [89], EL222 [90], and Magnets [91]. The light-regulated homodimer VVD has been used for light-induced gene repression (LEVI) [92] and expression (LexRO) [93]. Recently, Romano et al. reported a VVD-AraC fusion for a light-inducible system of the *araBAD* operon (BLADE) [94]. In 2017, the Liu research group reported photoactivatable T7 RNAP by creating fusion constructs of split T7 RNAP and VVD or its heterodimeric variant Magnets [95]. Later, the Khammash group reported an improved system using split T7 RNAP-Magnets fusions with inducible promoters (Opto-T7RNAP) [96]. In 2020, they demonstrated a new chemo-optogenetic control system with an improved dynamic range (from 65- to 700-fold) of Opto-T7RNAP by providing additional units of photodegradable anhydrotetracycline and a reverse Tet repressor [97]. The blue-light-induced homodimerization and DNA-binding properties of EL222 were utilized in the construction of a programmable transcriptional activator (pBLind) and repressor (pBLrep) in *E. coli* [98,99]. Recently, the Yuan research group reported a light-regulated CRISPR interference system (opto-CRISPRi) using EL222 and its cognate-binding sequence [100]. The opto-CRISPRi enabled the dynamic control of metabolic flux for optimal chemical production in *E. coli* by alternating the blue-light-induced growth phase and the dark-induced recovered production phase.

## 4. Conclusions and Perspectives

Modules for constructing a temperature-regulated gene expression system target all stages of gene expression, including replication (ts-replicon), transcription (ts-dimeric repressor and ts-RNAP), and translation (RNAT). As RNAT targets the late stage of gene expression, one can choose the RNAT system when a quick response is needed. Utilizing the ts-dimer module has advantages because of its relatively easy tunability at the working temperature [6,12] and modularity as a fusion partner, which broadens the scope of its applications. In contrast to the other ts modules, gene expression with the ts-replicon is irreversible. This limits the utility of the ts-replicon; however, it has advantages when permanent gene removal is required [18,21].

The LOV domain has proven useful in many light-controlled applications. However, there are some considerations when utilizing the LOV domains in optogenetics. One should consider the dynamic range and the leakiness of the system due to the imperfection in the association and dissociation dynamics of the LOV proteins [77]. Although flavin chromophores are naturally abundant, the amount in the cytosol may not be sufficient for generating fully loaded LOV domains [101]. This limits the efficiency of signal relay due to the unloaded non-functional apo-LOV proteins. This issue may be resolved by genetically encoding a photoswitchable amino acid that replaces flavin.

Systems employing non-invasive external signals, such as gene expression with TlpA-split T7 RNAP fusion [14] or Opto-T7RNAP [96], do not rely on chemical inducers, such as IPTG. This makes temperature- or light-regulated systems useful for reducing the cost of production when high-volume microbial cultures are used for large-scale chemical or protein production [7,99,102]. Although heat equilibrium and light penetration conditions need to be optimized at large industrial scales (>250 L), appropriate bioreactor designs, such as flow setups, may help in achieving success [103]. In this regard, the temperature- and light-controlled gene expression systems discussed in this review can be part of a valuable toolkit for the construction of microbial factories of chemicals or proteins required for industrial and biomedical applications.

## Data Availability

Not applicable.
